# Ethyl 3-(4-chloro­benzo­yl)-1-(4-chloro­benz­yl)-4-(4-chloro­phen­yl)-2,2-dioxo-3,4,6,7,8,8a-hexa­hydro-1*H*-pyrrolo­[2,1-*c*][1,4]thia­zine-1-carboxyl­ate

**DOI:** 10.1107/S1600536813009148

**Published:** 2013-04-13

**Authors:** A. Chitradevi, S. Athimoolam, S. Asath Bahadur, S. Indumathi, S. Perumal

**Affiliations:** a Department of Physics, Sri Subramanya College of Engineering & Technology, Palani 624 615, India; b Department of Physics, University College of Engineering, Nagercoil, Anna University, Tirunelveli Region, Nagercoil 629 004, India; c Department of Physics, Kalasalingam University, Krishnan Koil 626 190, India; d Department of Organic Chemistry, Madurai Kamaraj University, Madurai 625 021, India

## Abstract

In the title compound, C_30_H_28_Cl_3_NO_5_S, the pyrrolidine ring adopts an envelope conformation (with the N atom as the flap) and the thia­zine ring is in a distorted chair conformation. The mol­ecular structure shows three intra­molecular C—H⋯O inter­actions leading to self-associated ring *S*(6) and two *S*(7) motifs. In the crystal, the molecules are linked by C—H⋯O and C—H⋯Cl inter­actions. Two *R*_2_^2^(10) and one *R*_2_^2^(16) centrosymmetrically related ring motifs are observed in the unit cell and they are connected through *C*(6) and *C*(11) chain motifs extending along the *b* and *c* axes, respectively.

## Related literature

For the biological and pharmacological properties of thia­zine, pyrrolidine and pyrrolo­thia­zine compounds, see: Armenise *et al.* (1991 ▶, 1998 ▶); Hemming & Patel (2004 ▶); Koketsu *et al.* (2002 ▶); Kueh *et al.* (2003 ▶); Moriyama *et al.* (2004 ▶). For ring puckering analysis, see: Cremer & Pople (1975 ▶). For hydrogen-bonding inter­actions, see: Desiraju & Steiner (1999 ▶). For ring and chain motifs,see: Etter *et al.* (1990 ▶).
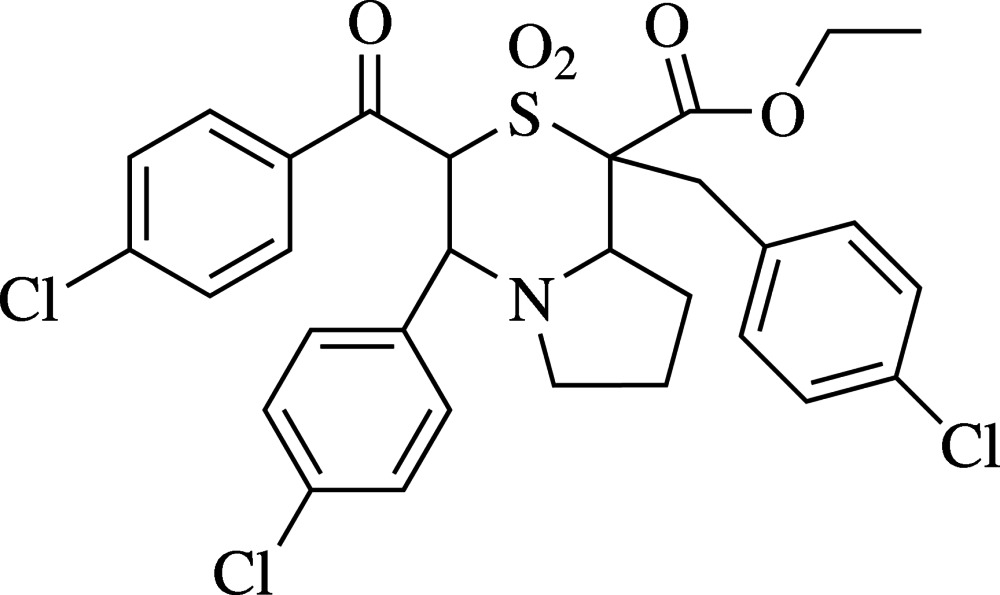


## Experimental

### 

#### Crystal data

C_30_H_28_Cl_3_NO_5_S
*M* *_r_* = 620.94
Monoclinic, 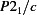

*a* = 14.0476 (8) Å
*b* = 17.1365 (9) Å
*c* = 13.8230 (8) Å
β = 115.893 (1)°
*V* = 2993.5 (3) Å^3^
*Z* = 4
Mo *K*α radiation
μ = 0.42 mm^−1^
*T* = 293 K
0.25 × 0.21 × 0.19 mm

#### Data collection

Bruker SMART APEX CCD area-detector diffractometer
28492 measured reflections
5280 independent reflections
4593 reflections with *I* > 2σ(*I*)
*R*_int_ = 0.022

#### Refinement

*R*[*F* ^2^ > 2σ(*F* ^2^)] = 0.048
*wR*(*F* ^2^) = 0.144
*S* = 1.04
5280 reflections
362 parameters
H-atom parameters constrained
Δρ_max_ = 0.62 e Å^−3^
Δρ_min_ = −0.47 e Å^−3^

### 

Data collection: *SMART* (Bruker, 2001 ▶); cell refinement: *SAINT* (Bruker, 2001 ▶); data reduction: *SAINT*; program(s) used to solve structure: *SHELXTL/PC* (Sheldrick, 2008 ▶); program(s) used to refine structure: *SHELXTL/PC*; molecular graphics: *PLATON* (Spek, 2009 ▶); software used to prepare material for publication: *SHELXTL/PC*.

## Supplementary Material

Crystal structure: contains datablock(s) global, I. DOI: 10.1107/S1600536813009148/hg5304sup1.cif

Structure factors: contains datablock(s) I. DOI: 10.1107/S1600536813009148/hg5304Isup2.hkl

Supplementary material file. DOI: 10.1107/S1600536813009148/hg5304Isup3.cml

Additional supplementary materials:  crystallographic information; 3D view; checkCIF report

## Figures and Tables

**Table 1:** Hydrogen-bond geometry (Å, °)

*D*—H⋯*A*	*D*—H	H⋯*A*	*D*⋯*A*	*D*—H⋯*A*
C6—H6*B*⋯O41	0.97	2.38	3.051 (3)	126
C37—H37⋯O2	0.93	2.53	3.213 (3)	131
C50—H50⋯O41	0.93	2.58	3.282 (4)	132
C6—H6*A*⋯O3^i^	0.97	2.70	3.582 (3)	151
C5—H5⋯O1^i^	0.98	2.51	3.320 (3)	140
C8—H8*B*⋯Cl3^ii^	0.97	2.86	3.678 (3)	143
C36—H36⋯O3^iii^	0.93	2.65	3.557 (3)	166
C42—H42*B*⋯O42^iv^	0.97	2.61	3.478 (5)	148

Symmetry codes: (i) 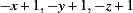; (ii) 
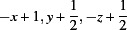
; (iii) 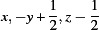; (iv) 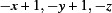.

**Fig. 1.**
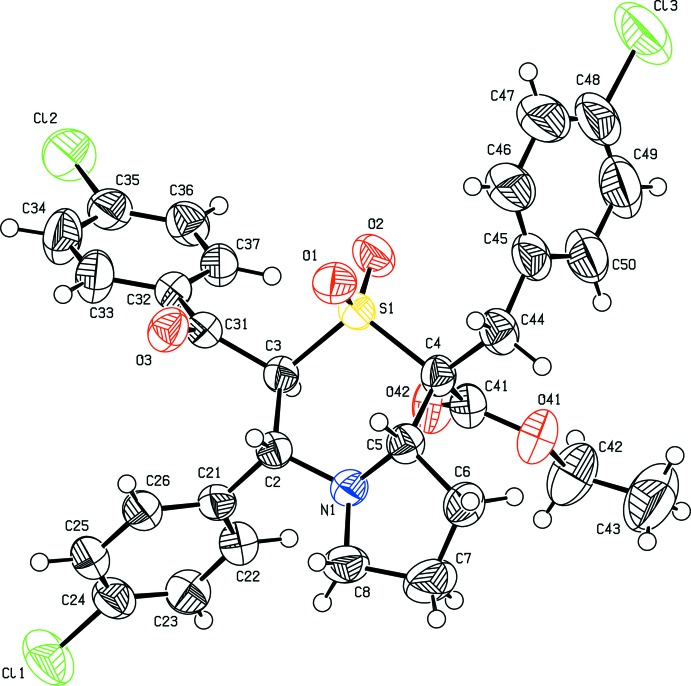
The molecular structure of the title compound with atom numbering scheme and 50% probability displacement ellipsoids. H-bonds are shown as dashed lines.

**Fig. 2.**
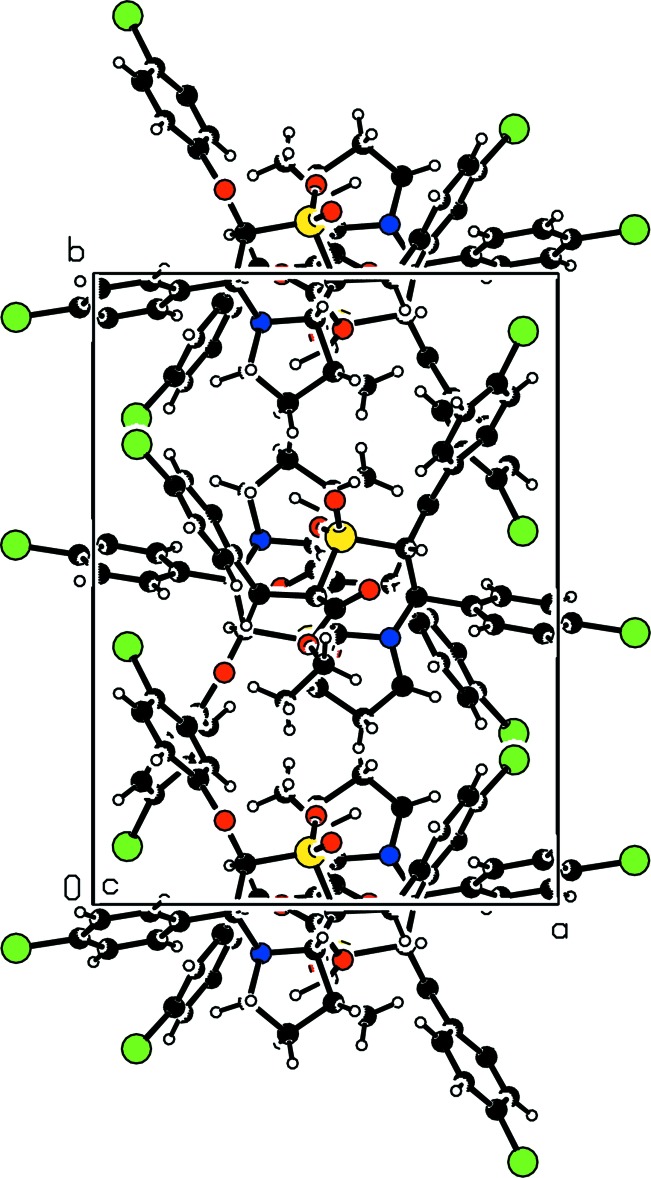
Packing diagram of the title compound viewed down the a-axis. H-bonds are shown as dashed lines.

## supplementary crystallographic information

### Comment

Thiazines occupy a unique place in medicinal chemistry since they show diverse biological properties such as antifungal, anti-inflammatory, anti-HIV, anti-psoriatic, sedative, neuroleptic, antitussive and anti-tubercular (Moriyama *et al.*, 2004; Koketsu *et al.*, 2002). In addition, compounds with a pyrrolidine sub-structure exhibit anti-tumour, analgesic, antidepressant, antihistaminic, anti-asthmatic and anti-Parkinson activities (Hemming & Patel, 2004; Kueh *et al.*, 2003). The pyrrolothiazine scaffold also shows anti-inflammatory, anti-fungal and anti-microbial activities (Armenise *et al.*, 1998; Armenise *et al.*, 1991).

The configuration and conformation of the title compound, (I) and the atom numbering scheme are shown in the *ORTEP* drawing (Fig. 1). The packing diagram of the title compound is shown in Fig. 2. The five-membered pyrrolidine ring is in envelope conformation [θ_2_ = 0.413 (2) Å, φ_2_ = 153 (1)°] and the six-membered thazine ring adopts a slightly distorted chair conformation [θ_2_ = 0.101 (2) Å, φ_2_ = 101 (1)° and θ_3_ = 0.661 (2) %A; Cremer & Pople, 1975]. The dihedral angle between the phenyl rings are observed to be 54.3 (1)°. The planes of the carboxylate groups are oriented with a dihedral angle of 22.3 (4)°.

The molecular structure conformation of the title compound features two intramolecular C—H···O interactions (Desiraju & Steiner, 1999). These intramolecular interactions are making self-associated ring S(6) and S(7) motifs (Table 1). Further, the crystal packing is stabilized through intermolecular C—H···O and C—H···Cl interactions. There are three centrosymmetric dimers are observed in the crystal, *viz*., two ring *R*_2_^2^(10) motifs and one *R*_2_^2^(16) motif (Etter *et al.*, 1990), formed through C5—H5···O1^i^, C42—H42···O42^i^ and C6—H6A···O3^iv^ interactions respectively. The C—H···Cl interactions connect the molecules along *b*-axis of unit cell and making a zigzag chain C(11) motif. Another chain C(6) motif is observed through C36—H36···O3^iii^ interaction, which is running along *c*-axis (For symmetry codes: see Table 1). Thus, the centrosymmetrically related dimers are tailored through these two chain motifs and the packing is stabilized.

### Experimental

A mixture of ethyl 2-[2-(4-chlorophenyl)-2-oxoethyl]sulfonylacetate (1.6 mmol), *p*-chloro benzaldehyde (3.2 mmol) and pyrrolidine (1.6 mmol) was dissolved in ethanol (10 ml), heated until the solution turned yellow and stirred at room temperature for 2–5 days. After completion of the reaction, the crude product was purified using flash column chromatography on silica gel (230–400 mesh) with petroleum ether and ethyl acetate mixture (95:5 *v*/*v*) as an eluent and subsequently it was recrystallized from ethanol.

### Refinement

All the H atoms were positioned geometrically and refined by the riding model approximation with d(C—H) = 0.93 - 0.98 Å and *U*_iso_(H) = 1.2-1.5*U*_eq_(C).

### Crystal data

**Table:**

C_30_H_28_Cl_3_NO_5_S	*F*(000) = 1288
*M**_r_* = 620.94	*D*_x_ = 1.378 Mg m^−^^3^
Monoclinic, *P*2_1_/*c*	Mo *K*α radiation, λ = 0.71073 Å
Hall symbol: -P 2ybc	Cell parameters from 2846 reflections
*a* = 14.0476 (8) Å	θ = 2.1–23.6°
*b* = 17.1365 (9) Å	µ = 0.42 mm^−^^1^
*c* = 13.8230 (8) Å	*T* = 293 K
β = 115.893 (1)°	Block, colourless
*V* = 2993.5 (3) Å^3^	0.25 × 0.21 × 0.19 mm
*Z* = 4	

### Data collection

**Table:**

Bruker SMART APEX CCD area-detector diffractometer	4593 reflections with *I* > 2σ(*I*)
Radiation source: fine-focus sealed tube	*R*_int_ = 0.022
Graphite monochromator	θ_max_ = 25.0°, θ_min_ = 2.0°
ω scans	*h* = −16→16
28492 measured reflections	*k* = −20→20
5280 independent reflections	*l* = −16→16

### Refinement

**Table:**

Refinement on *F*^2^	Primary atom site location: structure-invariant direct methods
Least-squares matrix: full	Secondary atom site location: difference Fourier map
*R*[*F*^2^ > 2σ(*F*^2^)] = 0.048	Hydrogen site location: inferred from neighbouring sites
*wR*(*F*^2^) = 0.144	H-atom parameters constrained
*S* = 1.04	*w* = 1/[σ^2^(*F*_o_^2^) + (0.0788*P*)^2^ + 1.5863*P*] where *P* = (*F*_o_^2^ + 2*F*_c_^2^)/3
5280 reflections	(Δ/σ)_max_ < 0.001
362 parameters	Δρ_max_ = 0.62 e Å^−^^3^
0 restraints	Δρ_min_ = −0.47 e Å^−^^3^

### Special details

**Table:**

Geometry. All e.s.d.'s (except the e.s.d. in the dihedral angle between two l.s. planes) are estimated using the full covariance matrix. The cell e.s.d.'s are taken into account individually in the estimation of e.s.d.'s in distances, angles and torsion angles; correlations between e.s.d.'s in cell parameters are only used when they are defined by crystal symmetry. An approximate (isotropic) treatment of cell e.s.d.'s is used for estimating e.s.d.'s involving l.s. planes.
Refinement. Refinement of *F*^2^ against ALL reflections. The weighted *R*-factor *wR* and goodness of fit *S* are based on *F*^2^, conventional *R*-factors *R* are based on *F*, with *F* set to zero for negative *F*^2^. The threshold expression of *F*^2^ > σ(*F*^2^) is used only for calculating *R*-factors(gt) *etc*. and is not relevant to the choice of reflections for refinement. *R*-factors based on *F*^2^ are statistically about twice as large as those based on *F*, and *R*- factors based on ALL data will be even larger.

### Fractional atomic coordinates and isotropic or equivalent isotropic displacement parameters (Å^2^)

**Table:**

	*x*	*y*	*z*	*U*_iso_*/*U*_eq_	
C2	0.30396 (17)	0.51264 (12)	0.30011 (17)	0.0434 (5)	
H2	0.3262	0.5069	0.3775	0.052*	
C3	0.32692 (17)	0.43589 (12)	0.25525 (18)	0.0421 (5)	
H3	0.2936	0.4386	0.1765	0.051*	
C4	0.52037 (17)	0.51148 (13)	0.28742 (17)	0.0462 (5)	
C5	0.47658 (18)	0.57139 (13)	0.34189 (18)	0.0464 (5)	
H5	0.4931	0.5526	0.4145	0.056*	
C6	0.5171 (2)	0.65580 (14)	0.3507 (2)	0.0581 (6)	
H6A	0.5623	0.6692	0.4250	0.070*	
H6B	0.5567	0.6626	0.3088	0.070*	
C7	0.4188 (3)	0.70537 (18)	0.3067 (4)	0.0956 (12)	
H7A	0.4288	0.7520	0.3499	0.115*	
H7B	0.4001	0.7206	0.2331	0.115*	
C8	0.3344 (2)	0.65402 (14)	0.3125 (2)	0.0639 (7)	
H8A	0.3390	0.6528	0.3845	0.077*	
H8B	0.2642	0.6709	0.2623	0.077*	
C21	0.18549 (17)	0.52703 (12)	0.24388 (18)	0.0451 (5)	
C22	0.13824 (19)	0.55639 (15)	0.1399 (2)	0.0539 (6)	
H22	0.1798	0.5666	0.1043	0.065*	
C23	0.0312 (2)	0.57072 (16)	0.0883 (2)	0.0597 (6)	
H23	0.0006	0.5914	0.0192	0.072*	
C24	−0.02958 (19)	0.55391 (16)	0.1408 (2)	0.0596 (6)	
C25	0.0141 (2)	0.52410 (16)	0.2430 (2)	0.0626 (7)	
H25	−0.0283	0.5125	0.2773	0.075*	
C26	0.12222 (19)	0.51156 (15)	0.2946 (2)	0.0546 (6)	
H26	0.1526	0.4924	0.3646	0.066*	
C31	0.27926 (17)	0.36683 (12)	0.29028 (18)	0.0441 (5)	
C32	0.22590 (18)	0.30213 (13)	0.21383 (18)	0.0470 (5)	
C33	0.1529 (2)	0.25797 (17)	0.2318 (2)	0.0703 (8)	
H33	0.1360	0.2714	0.2877	0.084*	
C34	0.1049 (3)	0.19463 (18)	0.1685 (3)	0.0780 (9)	
H34	0.0551	0.1656	0.1805	0.094*	
C35	0.1315 (2)	0.17463 (15)	0.0871 (2)	0.0607 (6)	
C36	0.2019 (2)	0.21813 (16)	0.0660 (2)	0.0589 (6)	
H36	0.2184	0.2044	0.0099	0.071*	
C37	0.24830 (19)	0.28249 (14)	0.12862 (19)	0.0521 (5)	
H37	0.2950	0.3130	0.1137	0.062*	
C41	0.47985 (19)	0.53106 (14)	0.16841 (19)	0.0514 (5)	
C42	0.5045 (3)	0.6192 (2)	0.0487 (2)	0.0892 (10)	
H42A	0.4357	0.6440	0.0231	0.107*	
H42B	0.5007	0.5780	−0.0010	0.107*	
C43	0.5867 (4)	0.6772 (3)	0.0586 (4)	0.1303 (18)	
H43A	0.5781	0.7233	0.0934	0.195*	
H43B	0.5797	0.6904	−0.0117	0.195*	
H43C	0.6556	0.6554	0.1005	0.195*	
C44	0.64259 (18)	0.50781 (16)	0.3518 (2)	0.0579 (6)	
H44A	0.6707	0.5581	0.3449	0.069*	
H44B	0.6589	0.5012	0.4271	0.069*	
C45	0.7021 (2)	0.44590 (17)	0.3230 (3)	0.0659 (7)	
C46	0.7454 (2)	0.3830 (2)	0.3891 (3)	0.0887 (10)	
H46	0.7330	0.3769	0.4495	0.106*	
C47	0.8069 (3)	0.3284 (2)	0.3689 (5)	0.1182 (16)	
H47	0.8355	0.2863	0.4150	0.142*	
C48	0.8248 (3)	0.3370 (3)	0.2819 (5)	0.1174 (18)	
C49	0.7820 (3)	0.3981 (3)	0.2116 (5)	0.1128 (15)	
H49	0.7937	0.4029	0.1507	0.135*	
C50	0.7208 (3)	0.4527 (2)	0.2339 (3)	0.0890 (10)	
H50	0.6920	0.4946	0.1874	0.107*	
N1	0.36103 (15)	0.57763 (10)	0.28163 (15)	0.0454 (4)	
S1	0.46708 (4)	0.41746 (3)	0.30207 (5)	0.04713 (18)	
Cl1	−0.16584 (6)	0.56953 (6)	0.07504 (8)	0.0933 (3)	
Cl2	0.07475 (7)	0.09158 (5)	0.01151 (8)	0.0880 (3)	
Cl3	0.90614 (12)	0.27103 (8)	0.2570 (2)	0.1971 (10)	
O1	0.51209 (14)	0.40207 (10)	0.41537 (14)	0.0607 (4)	
O2	0.47986 (14)	0.35984 (11)	0.23381 (16)	0.0647 (5)	
O3	0.28270 (14)	0.36726 (10)	0.37925 (13)	0.0571 (4)	
O41	0.53708 (16)	0.58805 (11)	0.15630 (14)	0.0672 (5)	
O42	0.40585 (15)	0.50130 (12)	0.09663 (14)	0.0698 (5)	

### Atomic displacement parameters (Å^2^)

**Table:**

	*U*^11^	*U*^22^	*U*^33^	*U*^12^	*U*^13^	*U*^23^
C2	0.0436 (11)	0.0422 (11)	0.0471 (11)	−0.0010 (9)	0.0222 (9)	−0.0014 (9)
C3	0.0413 (11)	0.0399 (11)	0.0474 (11)	−0.0022 (9)	0.0215 (9)	−0.0018 (9)
C4	0.0414 (11)	0.0481 (12)	0.0507 (12)	−0.0082 (9)	0.0216 (10)	−0.0057 (9)
C5	0.0440 (12)	0.0467 (12)	0.0465 (12)	−0.0054 (9)	0.0179 (9)	−0.0058 (9)
C6	0.0556 (14)	0.0497 (13)	0.0667 (15)	−0.0133 (11)	0.0247 (12)	−0.0127 (11)
C7	0.0680 (19)	0.0485 (16)	0.148 (3)	−0.0096 (14)	0.027 (2)	0.0009 (18)
C8	0.0571 (15)	0.0415 (12)	0.0856 (18)	0.0015 (11)	0.0243 (13)	−0.0055 (12)
C21	0.0455 (11)	0.0393 (11)	0.0518 (12)	0.0002 (9)	0.0225 (10)	−0.0037 (9)
C22	0.0509 (13)	0.0583 (14)	0.0554 (13)	−0.0026 (11)	0.0259 (11)	−0.0010 (11)
C23	0.0565 (15)	0.0637 (15)	0.0526 (14)	0.0005 (12)	0.0181 (11)	0.0019 (11)
C24	0.0437 (13)	0.0611 (15)	0.0692 (16)	0.0050 (11)	0.0202 (12)	−0.0023 (12)
C25	0.0525 (14)	0.0666 (16)	0.0780 (17)	0.0067 (12)	0.0372 (13)	0.0091 (13)
C26	0.0524 (13)	0.0561 (14)	0.0605 (14)	0.0065 (11)	0.0296 (11)	0.0077 (11)
C31	0.0434 (11)	0.0423 (11)	0.0506 (12)	0.0030 (9)	0.0241 (9)	0.0027 (9)
C32	0.0462 (12)	0.0414 (11)	0.0558 (13)	−0.0014 (9)	0.0245 (10)	0.0004 (9)
C33	0.0788 (19)	0.0686 (17)	0.0830 (18)	−0.0229 (15)	0.0534 (16)	−0.0172 (14)
C34	0.080 (2)	0.0710 (18)	0.099 (2)	−0.0318 (16)	0.0541 (18)	−0.0211 (16)
C35	0.0526 (14)	0.0492 (13)	0.0745 (16)	−0.0042 (11)	0.0224 (12)	−0.0125 (12)
C36	0.0558 (14)	0.0625 (15)	0.0603 (14)	−0.0016 (12)	0.0272 (12)	−0.0124 (12)
C37	0.0509 (13)	0.0534 (13)	0.0568 (13)	−0.0055 (10)	0.0281 (11)	−0.0031 (11)
C41	0.0499 (13)	0.0566 (13)	0.0526 (13)	−0.0084 (11)	0.0269 (11)	−0.0071 (11)
C42	0.117 (3)	0.092 (2)	0.0600 (17)	−0.030 (2)	0.0404 (18)	0.0024 (16)
C43	0.130 (4)	0.126 (4)	0.130 (4)	−0.023 (3)	0.052 (3)	0.046 (3)
C44	0.0408 (12)	0.0670 (16)	0.0617 (14)	−0.0076 (11)	0.0186 (11)	−0.0077 (12)
C45	0.0390 (12)	0.0688 (17)	0.0889 (19)	−0.0104 (12)	0.0270 (13)	−0.0145 (14)
C46	0.0550 (17)	0.085 (2)	0.119 (3)	0.0066 (16)	0.0307 (17)	0.004 (2)
C47	0.064 (2)	0.080 (3)	0.207 (5)	0.0059 (18)	0.055 (3)	−0.001 (3)
C48	0.064 (2)	0.074 (2)	0.235 (6)	−0.0205 (18)	0.084 (3)	−0.046 (3)
C49	0.098 (3)	0.104 (3)	0.180 (5)	−0.026 (2)	0.101 (3)	−0.043 (3)
C50	0.075 (2)	0.090 (2)	0.126 (3)	−0.0059 (17)	0.066 (2)	−0.014 (2)
N1	0.0441 (10)	0.0391 (9)	0.0520 (10)	−0.0027 (7)	0.0200 (8)	−0.0019 (8)
S1	0.0422 (3)	0.0435 (3)	0.0578 (4)	0.0003 (2)	0.0238 (3)	−0.0024 (2)
Cl1	0.0474 (4)	0.1287 (8)	0.0944 (6)	0.0176 (4)	0.0223 (4)	0.0155 (5)
Cl2	0.0776 (5)	0.0677 (5)	0.1101 (7)	−0.0189 (4)	0.0330 (5)	−0.0361 (4)
Cl3	0.1240 (10)	0.1021 (9)	0.429 (3)	−0.0200 (7)	0.1794 (16)	−0.0818 (13)
O1	0.0545 (10)	0.0600 (10)	0.0626 (10)	0.0061 (8)	0.0209 (8)	0.0127 (8)
O2	0.0539 (10)	0.0556 (10)	0.0929 (13)	−0.0025 (8)	0.0398 (9)	−0.0198 (9)
O3	0.0714 (11)	0.0530 (9)	0.0562 (10)	−0.0058 (8)	0.0363 (9)	0.0006 (7)
O41	0.0766 (12)	0.0707 (12)	0.0566 (10)	−0.0256 (10)	0.0314 (9)	−0.0020 (8)
O42	0.0675 (11)	0.0886 (14)	0.0498 (9)	−0.0284 (10)	0.0224 (9)	−0.0113 (9)

### Geometric parameters (Å, º)

**Table:**

C2—N1	1.459 (3)	C32—C33	1.382 (3)
C2—C21	1.518 (3)	C32—C37	1.387 (3)
C2—C3	1.547 (3)	C33—C34	1.372 (4)
C2—H2	0.9800	C33—H33	0.9300
C3—C31	1.538 (3)	C34—C35	1.375 (4)
C3—S1	1.812 (2)	C34—H34	0.9300
C3—H3	0.9800	C35—C36	1.368 (4)
C4—C41	1.525 (3)	C35—Cl2	1.739 (3)
C4—C5	1.551 (3)	C36—C37	1.378 (3)
C4—C44	1.552 (3)	C36—H36	0.9300
C4—S1	1.825 (2)	C37—H37	0.9300
C5—N1	1.469 (3)	C41—O42	1.193 (3)
C5—C6	1.540 (3)	C41—O41	1.321 (3)
C5—H5	0.9800	C42—O41	1.453 (3)
C6—C7	1.505 (4)	C42—C43	1.483 (5)
C6—H6A	0.9700	C42—H42A	0.9700
C6—H6B	0.9700	C42—H42B	0.9700
C7—C8	1.505 (4)	C43—H43A	0.9600
C7—H7A	0.9700	C43—H43B	0.9600
C7—H7B	0.9700	C43—H43C	0.9600
C8—N1	1.475 (3)	C44—C45	1.508 (4)
C8—H8A	0.9700	C44—H44A	0.9700
C8—H8B	0.9700	C44—H44B	0.9700
C21—C26	1.377 (3)	C45—C46	1.372 (5)
C21—C22	1.388 (3)	C45—C50	1.372 (5)
C22—C23	1.376 (4)	C46—C47	1.383 (5)
C22—H22	0.9300	C46—H46	0.9300
C23—C24	1.370 (4)	C47—C48	1.340 (7)
C23—H23	0.9300	C47—H47	0.9300
C24—C25	1.370 (4)	C48—C49	1.375 (7)
C24—Cl1	1.744 (2)	C48—Cl3	1.745 (4)
C25—C26	1.384 (3)	C49—C50	1.393 (5)
C25—H25	0.9300	C49—H49	0.9300
C26—H26	0.9300	C50—H50	0.9300
C31—O3	1.209 (3)	S1—O2	1.4302 (18)
C31—C32	1.489 (3)	S1—O1	1.4343 (18)
			
N1—C2—C21	110.48 (18)	C37—C32—C31	123.6 (2)
N1—C2—C3	110.62 (17)	C34—C33—C32	120.9 (3)
C21—C2—C3	107.86 (17)	C34—C33—H33	119.5
N1—C2—H2	109.3	C32—C33—H33	119.5
C21—C2—H2	109.3	C33—C34—C35	119.1 (3)
C3—C2—H2	109.3	C33—C34—H34	120.5
C31—C3—C2	109.41 (17)	C35—C34—H34	120.5
C31—C3—S1	107.94 (14)	C36—C35—C34	121.3 (2)
C2—C3—S1	112.91 (14)	C36—C35—Cl2	120.2 (2)
C31—C3—H3	108.8	C34—C35—Cl2	118.6 (2)
C2—C3—H3	108.8	C35—C36—C37	119.3 (2)
S1—C3—H3	108.8	C35—C36—H36	120.3
C41—C4—C5	109.74 (19)	C37—C36—H36	120.3
C41—C4—C44	115.21 (19)	C36—C37—C32	120.5 (2)
C5—C4—C44	108.81 (18)	C36—C37—H37	119.7
C41—C4—S1	109.64 (15)	C32—C37—H37	119.7
C5—C4—S1	105.17 (14)	O42—C41—O41	124.6 (2)
C44—C4—S1	107.77 (17)	O42—C41—C4	125.6 (2)
N1—C5—C6	104.79 (18)	O41—C41—C4	109.77 (19)
N1—C5—C4	110.32 (17)	O41—C42—C43	105.5 (3)
C6—C5—C4	116.68 (19)	O41—C42—H42A	110.6
N1—C5—H5	108.3	C43—C42—H42A	110.6
C6—C5—H5	108.3	O41—C42—H42B	110.6
C4—C5—H5	108.3	C43—C42—H42B	110.6
C7—C6—C5	104.8 (2)	H42A—C42—H42B	108.8
C7—C6—H6A	110.8	C42—C43—H43A	109.5
C5—C6—H6A	110.8	C42—C43—H43B	109.5
C7—C6—H6B	110.8	H43A—C43—H43B	109.5
C5—C6—H6B	110.8	C42—C43—H43C	109.5
H6A—C6—H6B	108.9	H43A—C43—H43C	109.5
C8—C7—C6	104.7 (2)	H43B—C43—H43C	109.5
C8—C7—H7A	110.8	C45—C44—C4	118.6 (2)
C6—C7—H7A	110.8	C45—C44—H44A	107.7
C8—C7—H7B	110.8	C4—C44—H44A	107.7
C6—C7—H7B	110.8	C45—C44—H44B	107.7
H7A—C7—H7B	108.9	C4—C44—H44B	107.7
N1—C8—C7	101.5 (2)	H44A—C44—H44B	107.1
N1—C8—H8A	111.5	C46—C45—C50	117.5 (3)
C7—C8—H8A	111.5	C46—C45—C44	120.8 (3)
N1—C8—H8B	111.5	C50—C45—C44	121.5 (3)
C7—C8—H8B	111.5	C45—C46—C47	122.1 (4)
H8A—C8—H8B	109.3	C45—C46—H46	118.9
C26—C21—C22	118.3 (2)	C47—C46—H46	118.9
C26—C21—C2	121.0 (2)	C48—C47—C46	119.0 (4)
C22—C21—C2	120.7 (2)	C48—C47—H47	120.5
C23—C22—C21	121.4 (2)	C46—C47—H47	120.5
C23—C22—H22	119.3	C47—C48—C49	121.5 (4)
C21—C22—H22	119.3	C47—C48—Cl3	120.0 (5)
C24—C23—C22	118.7 (2)	C49—C48—Cl3	118.5 (5)
C24—C23—H23	120.6	C48—C49—C50	118.5 (4)
C22—C23—H23	120.6	C48—C49—H49	120.7
C23—C24—C25	121.5 (2)	C50—C49—H49	120.7
C23—C24—Cl1	119.2 (2)	C45—C50—C49	121.3 (4)
C25—C24—Cl1	119.4 (2)	C45—C50—H50	119.4
C24—C25—C26	119.1 (2)	C49—C50—H50	119.4
C24—C25—H25	120.5	C2—N1—C5	113.62 (17)
C26—C25—H25	120.5	C2—N1—C8	113.53 (18)
C21—C26—C25	121.0 (2)	C5—N1—C8	104.91 (17)
C21—C26—H26	119.5	O2—S1—O1	118.10 (12)
C25—C26—H26	119.5	O2—S1—C3	108.33 (10)
O3—C31—C32	120.7 (2)	O1—S1—C3	108.31 (10)
O3—C31—C3	119.06 (19)	O2—S1—C4	111.14 (11)
C32—C31—C3	120.19 (19)	O1—S1—C4	106.08 (11)
C33—C32—C37	118.8 (2)	C3—S1—C4	103.94 (10)
C33—C32—C31	117.6 (2)	C41—O41—C42	117.7 (2)
			
N1—C2—C3—C31	171.76 (17)	S1—C4—C41—O42	17.2 (3)
C21—C2—C3—C31	−67.3 (2)	C5—C4—C41—O41	80.3 (2)
N1—C2—C3—S1	51.5 (2)	C44—C4—C41—O41	−42.9 (3)
C21—C2—C3—S1	172.46 (14)	S1—C4—C41—O41	−164.69 (17)
C41—C4—C5—N1	51.9 (2)	C41—C4—C44—C45	−64.2 (3)
C44—C4—C5—N1	178.80 (18)	C5—C4—C44—C45	172.1 (2)
S1—C4—C5—N1	−66.0 (2)	S1—C4—C44—C45	58.5 (3)
C41—C4—C5—C6	−67.5 (2)	C4—C44—C45—C46	−107.4 (3)
C44—C4—C5—C6	59.4 (3)	C4—C44—C45—C50	76.8 (3)
S1—C4—C5—C6	174.66 (17)	C50—C45—C46—C47	0.8 (5)
N1—C5—C6—C7	7.3 (3)	C44—C45—C46—C47	−175.1 (3)
C4—C5—C6—C7	129.7 (3)	C45—C46—C47—C48	−0.1 (6)
C5—C6—C7—C8	19.4 (3)	C46—C47—C48—C49	−1.0 (6)
C6—C7—C8—N1	−38.9 (3)	C46—C47—C48—Cl3	177.6 (3)
N1—C2—C21—C26	−136.5 (2)	C47—C48—C49—C50	1.4 (6)
C3—C2—C21—C26	102.5 (2)	Cl3—C48—C49—C50	−177.3 (3)
N1—C2—C21—C22	43.3 (3)	C46—C45—C50—C49	−0.4 (5)
C3—C2—C21—C22	−77.7 (2)	C44—C45—C50—C49	175.5 (3)
C26—C21—C22—C23	0.7 (4)	C48—C49—C50—C45	−0.7 (6)
C2—C21—C22—C23	−179.1 (2)	C21—C2—N1—C5	174.57 (17)
C21—C22—C23—C24	−1.3 (4)	C3—C2—N1—C5	−66.1 (2)
C22—C23—C24—C25	0.6 (4)	C21—C2—N1—C8	54.8 (2)
C22—C23—C24—Cl1	−178.4 (2)	C3—C2—N1—C8	174.15 (19)
C23—C24—C25—C26	0.7 (4)	C6—C5—N1—C2	−156.72 (19)
Cl1—C24—C25—C26	179.8 (2)	C4—C5—N1—C2	76.9 (2)
C22—C21—C26—C25	0.7 (4)	C6—C5—N1—C8	−32.1 (2)
C2—C21—C26—C25	−179.5 (2)	C4—C5—N1—C8	−158.5 (2)
C24—C25—C26—C21	−1.4 (4)	C7—C8—N1—C2	168.8 (2)
C2—C3—C31—O3	−37.8 (3)	C7—C8—N1—C5	44.2 (3)
S1—C3—C31—O3	85.4 (2)	C31—C3—S1—O2	75.70 (17)
C2—C3—C31—C32	139.7 (2)	C2—C3—S1—O2	−163.24 (16)
S1—C3—C31—C32	−97.0 (2)	C31—C3—S1—O1	−53.52 (17)
O3—C31—C32—C33	20.7 (3)	C2—C3—S1—O1	67.55 (17)
C3—C31—C32—C33	−156.8 (2)	C31—C3—S1—C4	−166.03 (14)
O3—C31—C32—C37	−157.0 (2)	C2—C3—S1—C4	−44.96 (17)
C3—C31—C32—C37	25.5 (3)	C41—C4—S1—O2	48.18 (19)
C37—C32—C33—C34	1.5 (4)	C5—C4—S1—O2	166.11 (15)
C31—C32—C33—C34	−176.3 (3)	C44—C4—S1—O2	−77.93 (18)
C32—C33—C34—C35	0.9 (5)	C41—C4—S1—O1	177.75 (15)
C33—C34—C35—C36	−2.2 (5)	C5—C4—S1—O1	−64.32 (16)
C33—C34—C35—Cl2	177.0 (3)	C44—C4—S1—O1	51.64 (17)
C34—C35—C36—C37	1.0 (4)	C41—C4—S1—C3	−68.15 (17)
Cl2—C35—C36—C37	−178.2 (2)	C5—C4—S1—C3	49.78 (16)
C35—C36—C37—C32	1.5 (4)	C44—C4—S1—C3	165.74 (15)
C33—C32—C37—C36	−2.7 (4)	O42—C41—O41—C42	3.1 (4)
C31—C32—C37—C36	174.9 (2)	C4—C41—O41—C42	−175.0 (3)
C5—C4—C41—O42	−97.8 (3)	C43—C42—O41—C41	−175.9 (3)
C44—C4—C41—O42	139.0 (3)		

### Hydrogen-bond geometry (Å, º)

**Table:**

*D*—H···*A*	*D*—H	H···*A*	*D*···*A*	*D*—H···*A*
C6—H6*B*···O41	0.97	2.38	3.051 (3)	126
C37—H37···O2	0.93	2.53	3.213 (3)	131
C50—H50···O41	0.93	2.58	3.282 (4)	132
C6—H6*A*···O3^i^	0.97	2.70	3.582 (3)	151
C5—H5···O1^i^	0.98	2.51	3.320 (3)	140
C8—H8*B*···Cl3^ii^	0.97	2.86	3.678 (3)	143
C36—H36···O3^iii^	0.93	2.65	3.557 (3)	166
C42—H42*B*···O42^iv^	0.97	2.61	3.478 (5)	148

Symmetry codes: (i) −*x*+1, −*y*+1, −*z*+1; (ii) −*x*+1, *y*+1/2, −*z*+1/2; (iii) *x*, −*y*+1/2, *z*−1/2; (iv) −*x*+1, −*y*+1, −*z*.
